# DEVELOPING A GERIATRIC EMERGENCY DEPARTMENT BY FOCUSING ON THE THREE PS: PEOPLE, PROCESSES, AND PLACE

**DOI:** 10.1093/geroni/igad104.2061

**Published:** 2023-12-21

**Authors:** John Schumacher, Don Melady

**Affiliations:** University of Maryland, Baltimore County, Baltimore, Maryland, United States; University of Toronto, Toronto, Ontario, Canada

## Abstract

Older patients’ emergency department (ED) visits in the U.S. rose a surprising 30% during the period 2015-2019 to about 28 million visits annually. Many EDs are now examining emerging geriatric emergency department (GED) models of care to address the care needs of this population. This paper provides a practical overview of how to develop a GED and introduces the 3 Ps model focusing attention on the ED’s People, Processes, and the Place thereby providing a clear framework for practical GED development. Key resources facilitating GED development includes: the national GED Collaborative (GEDC) organization, the nationally endorsed GED Guidelines, and resources associated with the Geriatric Emergency Department Accreditation process. Core clinical elements needed to create a GED’s operations are found in its care processes including: 1) General approaches to the patient; 2) Screening for high-risk conditions; 3) Enhanced assessment for older patients; 4) potential workflow alterations; and 5) Attention to transitions into and out of the ED. The paper concludes by provides additional practical guidance to EDs seeking to enhance the ED experience of older people and improve the quality of their outcomes.

